# Supplementary material for: Non-Fullerene Small Molecule Electron-Transporting Materials for Efficient p-i-n Perovskite Solar Cells
Source: Nanomaterials (Basel). 2020 May 31;10(6):1082. doi: 10.3390/nano10061082 (PMC7353412; doi:10.3390/nano10061082)
Supplement: Supplementary file 1 [file nanomaterials-10-01082-s001.pdf]

nanomaterials

MDPI

Article

Non-Fullerene Small Molecule Electron-Transporting Materials for Efficient p-i-n Perovskite Solar Cells

Da-Seul Choi, Sung-Nam Kwon * and Seok-In Na *

Graduate School of Flexible and Printable Electronics, LANL-JBNU Engineering Institute-Korea, Jeonbuk National University, 567 Baekje-daero, Deokjin-gu, Jeonju-si 54896, Korea; ektmf6175@jbnu.ac.kr
* Correspondence: dasom2u@jbnu.ac.kr (S.-N.K); nsi@jbnu.ac.kr (S.-I.N)

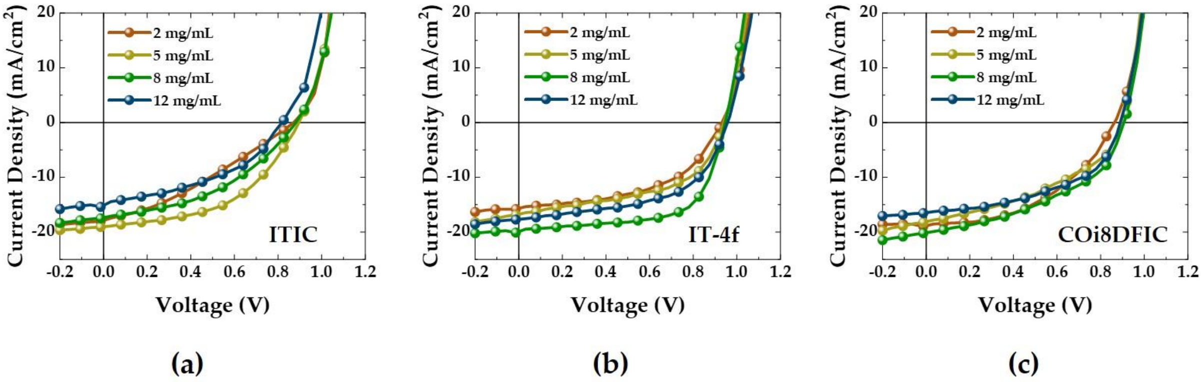

Figure S1. Current density–voltage (J–V) curves of the PSC according to the concentration change of (**a**) ITIC, (**b**) IT-4f, and (**c**) COi8DFIC.

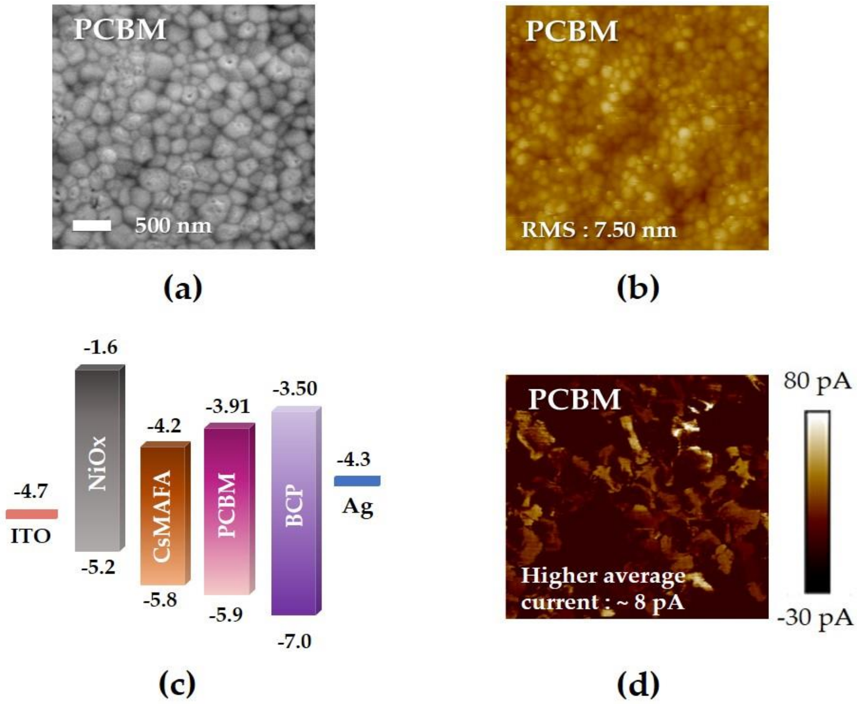

Figure S2. (**a**) Top-view SEM and (**b**) AFM images (scale: 5 × 5 μm) of PCBM films coated on the ITO/NiOx/perovskite layer, (**c**) Schematic energy level diagram of the perovskite and PCBM ETLs, (**d**) C-AFM image (scale: 2 × 2 μm) of PCBM film.

Table S1. Fitting parameters of bi-exponential decay function in transient photovoltage measurement.

Film	Amplitude A_1	Decay time τ_1 (μs)	Amplitude A_2	Decay time τ_2 (μs)	Average decay time τ_w (μs) [a]
ITIC	0.95	176	0.08	1310	263
IT-4f	0.81	289	0.17	2410	649
COi8DFIC	0.84	173	0.08	1110	257

[a] Average decay time is calculated according to the equation: $\tau_w = (A_1\tau_1+A_2\tau_2)/(A_1+A_2)$.

Table S2. Fitting parameters of bi-exponential decay function in transient photocurrent measurement.

Film	Amplitude A_1	Decay time τ_1 (μs)	Amplitude A_2	Decay time τ_2 (μs)	Average decay time τ_w (μs) [a]
ITIC	1.72	0.55	0.66	0.55	0.55
IT-4f	2.32	0.43	0.21	0.43	0.43
COi8DFIC	1.72	0.56	0.43	0.56	0.56

[a] Average decay time is calculated according to the equation: $\tau_w = (A_1\tau_1+A_2\tau_2)/(A_1+A_2)$.
